# Medica Sacra; or, Short Expositions of the Most Important Diseases Mentioned in Scripture

**Published:** 1834-07-01

**Authors:** 


### Medica Sacra; or short Expositions of the most Important Diseases mentioned in the Sacred Writings. By *Thomas Shapter*, M.D. &c. Octavo, pp. 191. Longman and Co. 1834.

As human nature—at least since the flood of Noah—was probably the same as in the present day, there can be little doubt that some—perhaps many, of the diseases to which flesh is heir, occurred in the time of Moses, not essentially different (though modified) from those which we now perceive and treat. Life lingered out then, as now, to the extent of " threescore years and ten"—more or less—but, as they had not the lights of modern pathology, and the discrimination of modern nosology, we can only guess at the particular diseases by which existence was curtailed four or five thousand years ago, by the somewhat vague descriptions contained in the oldest of human writings. It is, of course, more curious than useful to trace these analogies or verisimilitudes—but, in the dearth or profusion (whichever it may be called) of modern novelty, the researches of Dr. Shapter will not prove an unacceptable variety. There is an interest attached to antiquity, which the most philosophic mind cannot divest itself of—and, when the Sacred Writings are in question, the interest is doubled. It will hardly be doubted that we have witnessed a considerable number of dissections of ovarian disease; yet the presentation of a diseased ovarium in an Egyptian mummy, which we saw in the library of our friend Dr. Granville, excited more vivid emotions in the mind than any *post-mortem* investigation which we remember. We cannot give any philosophical or satisfactory reason for this—but so it was—and so it ever will be.

The Jews and the Egyptians have had a great advantage over us moderns. The Sacred Writings of the former will descend, in secula seculorum; whereas, both the *sacred* and the profane writings of the present day have little chance of preservation three or four thousand years hence. The Egyptians took such especial care of the lifeless body, that the mummy comes down to us nearly as fresh as when first swathed in bitumened rollers, and thus specimens of morbid anatomy are preserved beyond the power of wax or spirits in our choicest museums.

We have said that the investigations of Dr. Shapter are more curious than useful—more amusing than profitable. The same remark applies to his predecessor in the same walk—Dr. Mead. It is remarkable that this venerable physician not only veiled his Medica Sacra from vulgar eyes in the Latin garb, but anathematized the attempt to make it popular, by translation into English! It is difficult to conjecture the reason for this prohibition. Doubtless they had reference to some religious points of doctrine—probably to the notion, that the diseases mentioned in Scripture were miraculous or direct inflictions of the Deity—a doctrine, by the way, which is, even now, embraced by a large portion of society respecting the diseases by which we are afflicted. The cholera, for example, was considered by millions of people in these Isles, as a direct interference of the Almighty on a sinful world! It is hardly necessary to say, that if we apply this principle of causation to one disease, or to one epidemic, we must apply it to all—nor can the advocates of this doctrine appear very consistent, when they strenuously urge the physician to counteract the dispensations of Providence through the medium of drugs!

Our author looks upon the diseases described in Holy Writ as infirmities of Nature, resulting from natural causes, the same as at present, and he thinks that the investigation is interesting, as "shewing the height to which the knowledge of the phenomena attending the physical changes in man had arrived in early times, and the accuracy with which the various observations, opinions, and remarks relating to them are recorded."

The blood is called the life of the flesh (Genesis), and the heart the vessel from which proceed the issues of life (Leviticus), while the bones are considered as the tenement of these life-sources. The period of life is limited in Scripture to three or fourscore years, after which "strength is labour and sorrow." The approaches to age are marked in Scripture (Dr. S. thinks) by mental, rather than by corporeal defects.

"Remember now thy Creator in the days of thy youth, while the evil days come not, nor the years draw nigh when thou shalt say, I have no pleasure in them."* 7.

We agree with Lorinus rather than with Dr. Shapter. We think that, if he interpreted the Bible wrong, the Bible is right—and that if he construed it right, the Bible is wrong. We think the corporeal functions begin to fail before the mental faculties begin to decay. The corporeal fabric arrives at its full development before the mind attains its maximum of strength—and it feels the hand of time sooner than the immaterial tenant. Our belief, indeed, is, that the mental deteriorations are principally, if not wholly, *caused* by the material dilapidations, and consequently are posterior, in point of time, to these last. To this must be added, the bodily diseases to which we are liable throughout the whole range of life, and which, when they act at all on the mind, can only do so secondarily. The following passage bears strongly on a point which we have slightly mooted above.

"From the Sacred Writings we learn, that disease and death are the consequences or 'wages of sin,' and that both are of divine imposition, for 'with a great plague will the Lord smite;' and each may say to himself, 'I know THOU wilt bring me to death.'

So impressed were the Hebrews with a narrow superstitious mode of considering these subjects, that they would not permit the least idea of an influence from natural causes to intervene between their prejudices, and what should have been obvious to them by the tenor of the Mosaical law.

For it is there plainly shewn that the plague, though expressly stated to be of divine infliction, is to be treated under the sanction of God by human efforts and natural means, as is ordered of every thing in this world." 15.

* "Lorinus says, that these last words have a much more extensive meaning in the original;—that they relate to the capability of performing one's ordinary duties or business."

In respect to this "divine infliction," it is to be remembered that the ordinary style of oriental writing is often so redolent of figure and poetic illustration, as greatly to obscure the true meaning of the unadorned statement.

"Dr. Mead, in speaking of the plague, is quite of this opinion; 'for these evils and calamities (he observes) which come suddenly, and strike all with terror, are said sometimes to proceed from God; but which nevertheless have their origin in natural causes.'" 18.

If Moses really considered diseases as "divine inflictions," why did he prescribe the means of avoiding or curing them? Why did he prohibit blood—swine's flesh, &c. and also separation from the afflicted? Viewing the diseases of Scripture, therefore, as natural infirmities, and open to human investigation, the author proceeds to particularize them.

Leprosy naturally leads the van, not only on account of the frequency with which it is mentioned in the Holy Writings, but of the august personages who were afflicted with it. The author has expended great research, and much cogitation, on the analogies or identities of the ancient and the modern lepra; but we regret to say that we cannot transfer any thing useful from the author's to our own pages. These investigations, however, will prove more interesting to divines than to physicians—and more curious than useful to either. The same observations will apply to plague, and to boils and blains. The latter is conjectured by our author to be the same as our present variola. Should this be true, the origin of the disease is not a little curious.

"And they took the ashes of the furnace, and stood before Pharaoh, and Moses sprinkled it up towards Heaven; and it became a boil, breaking forth with blains upon man and upon beast."

How came it that the animal kingdom escaped this sixth plague of Egypt afterwards? Or was the cow-pox a remnant of this miraculous origin of the variolous poison? We doubt whether the author was quite prudent in meddling with these plagues.

"Of the ten plagues of Egypt, but one, properly speaking, comes now within my province, though I could derive some advantage to my argument in pointing out, that each of them would tend to prove the truth of my positions, that these afflictions are of *divine imposition*, and that they have their *origin* in, and are subject to, the ordinary laws, to which diseases are said to be subject in the present day." 110.

If they were of "divine imposition," how can they be said to have their origin in the "ordinary laws" of nature? If the sixth plague was a miracle, the "ashes from the furnace," thrown up into the air, could not surely be considered as an illustration of the ordinary laws of causation, according to to our present knowledge.

In the discussion on the demoniacs of the New Testament, our author conceives that the "true condition of the demoniac was lunacy," and that no supernatural agency is inferred—the figurative language of Jews and Greeks attributing mania, epilepsy, and even the higher degrees of rage and distraction, to evil demons. This is probably the true state of the case. When a miracle is stated in Scripture we ought to believe it, because the narrative is divine—or states divine things. The course of Nature is interrupted for the moment, and it is quite absurd to attempt to reconcile the ordinary laws of Nature with their miraculous suspension. What we contend for is, that miracles, and the necessity for miracles, having ceased, we ought not *now* to make every occurrence a miracle—to make every epidemic a dispensation of Providence, and every disease, a divine infliction on the individual for his sins!

The disease of JOB is associated with our earliest recollections. Some have doubted the existence of this personage; but Hugo Grotius appears to our author to have fully proved that JOB was a real person—that his sufferings and patience are a portion of his history, though, according to the custom of the times, it is poetically described. As he was not antediluvian, his age, of more than 200 years, is, we confess, a stumbling-block as to the reality of the individual. Hales places Job's trial 2130 years before the Christian æra.

Having suffered several severe moral ills, in the loss of children, Job became afflicted with boils from head to foot, which greatly disfigured him. His friends remained seven days and nights with him, during which they did not address him, his pain being so great, and his restlessness so much increased, that he spoke in wailing.

Dr. Mead thinks that the disease was elephantiasis; but there seems little reason for coming to this conclusion. Our author's conjecture is far more probable—namely, that it was small-pox.

The disease or disorder of King Saul appears to have been melancholia, rather than mania. It was occasioned by reverses of fortune, and cured by the harp of David.

The malady of Nebuchadnezzar is looked upon by our author as a species of monomania, in which the patient fancied himself an ox, and went into the fields to eat grass accordingly. King Jehoram, Dr. Shapter doubts not, laboured under chronic dysentery, "though some take it to be an hernia, others a falling of the anus, or fistula, &c." Epilepsy, like mania, is represented in Scripture as caused by the possession of evil spirits. It is needloss to comment on this doctrine.

As we said before, there is more curiosity than utility attached to this investigation, though it has exercised the pens and thoughts of such men as Mead, and other celebrated characters. We think the investigation fruitless—first, because it is by no means certain, that the diseases in the time of Moses were the same as they are now. We know that many have entirely disappeared—and that others, as syphilis, &c. have sprung up at comparatively recent periods. *Secondly*, because the statements in Scripture are so veiled in metaphorical language, that it is impossible to come to an accurate idea of the diseases they describe. *Thirdly*, because, if we could determine to an azimuth the sacred maladies, it would not advance either pathology or therapeutics.

